# Allee effects in an invasive social wasp: an experimental study in colonies of *Vespula germanica*

**DOI:** 10.1038/s41598-023-43527-7

**Published:** 2023-09-28

**Authors:** Romina Melo, Maité Masciocchi, Juan C. Corley

**Affiliations:** 1 Grupo de Ecología de Poblaciones de Insectos, IFAB (CONICET, INTA EEA Bariloche), Modesta Victoria 4450, 8400 Bariloche, Rio Negro Argentina; 2 Departamento de Ecología, Centro Regional Universitario Bariloche, Universidad Nacional Del Comahue, Quintral 1250, 8400 Bariloche, Argentina

**Keywords:** Ecology, Population dynamics, Entomology

## Abstract

Allee effects rely on the existence of mutually beneficial intraspecific interactions that increase individual fitness and per capita growth rate, as the number of individuals in a population or group increases. When the number of individuals falls below a given number, the success of a group or population drops. Social insects heavily rely on cooperation between individuals for various tasks such as foraging and breeding. In this study, we experimentally explored component Allee effects and the underlying mechanisms in colonies of the social wasp *Vespula germanica*. After the removal of workers, we counted the number of gynes produced, estimated the body mass index as a proxy of their quality, and registered the protein-food collected by foragers in colonies. Our research revealed a correlation between the decrease in worker population and a subsequent decrease in the production of gynes. However, the removal of workers did not impact the quality of the produced gynes or the quantity of protein-food collected by the colonies. These findings highlight the crucial role of the worker caste in the success of eusocial species and suggested an ability of workers to perform multiple tasks that enable colonies to respond to disturbances. Additionally, our study provides the first evidence of Allee effects at the colony level of *V. germanica*, with potential practical implications for managing this invasive species.

## Introduction

The positive correlation between per capita population growth rate or individual fitness and population size is known as the “Allee effect”^1,2^. This positive density-dependence relies on the existence of mutually beneficial interactions between individuals of the same group or population. This phenomenon can be differentiated into two types: “demographic” and “component” Allee effects. Demographic Allee effects refer to the relationship between the size of a group or population and growth rate, whereas component Allee effects refers to the relationship between group or population size and the fitness of its individuals^3–5^. An important caveat is that a demographic Allee effect is always a consequence of at least one component Allee effect, but the existence of a component Allee effect does not always lead to demographic effects^5,6^. Even though Allee effects have proven difficult to detect empirically, they have been observed in plant and animal species, being especially common in terrestrial arthropods^7^.

Allee effects have been proposed as a phenomenon at the population level, however, in social species the population dynamics is influenced by another level of organization: the social group^3,8^. In social species, individuals group to maximize their fitness, so most cooperative interactions occur between individuals of the same group, while reproduction and competitive interactions occur between individuals of different groups. Likewise, the benefits of these social or cooperative behaviours depend on the size of the group. For example, in the African wild dog *Lycaon pictus*, the number of pups per year and their survival is higher in bug groups since because pups receive more protection and food from helpers compared to small groups^9^. Since individuals aggregate or form aggregations to maximize their fitness, it has been suggested that Allee effects could occur at different levels of organization (population or group) depending on which benefit is greater^8^.

Component Allee effects are the result of any mechanism that generates a positive density dependence in some component of fitness. Well known examples are mate finding, predator dilution, chemical and physical facilitation, and some social interactions^5,7^. In social species where numerous activities are performed cooperatively (breeding, hunting, foraging, feeding, and protection from predators), component Allee effects may relate directly to social interactions. For example, in the African wild dog *Lycaon pictus,* individuals benefit from cooperative hunting and breeding and some components of individual fitness (i.e., litter size or pup survival) are positively related to pack size^9^. Similarly, in the social spider *Anelosimus eximius,* members of a colony cooperate to capture prey during feeding and brood care; consequently, the mean number of eggs and the offspring survival of a spider increases with colony size^10^. Luque et al*.*^11^ studied component Allee effects at the colony level of the invasive Argentine ant (*Linepithema humile*) and showed unambiguously, that the number of workers increased the fitness of queens. From the standpoint of cooperation, any increase in sociability could be translated into an increase in susceptibility to Allee effects^3,5^. However, only few studies have focused on the existence and strength of Allee effects in social insects^7,12,13^.

Cooperation among individuals is a defining character of social insects^14^. Individuals of the same colony operate and reproduce as a unit and survive only by living in a common nest^15,16^. Ants, some bees, and wasps are emblematic examples of eusociality and active cooperation. Individuals within a nest have different roles, with a few being capable of mating (the reproductive caste) while the rest (the worker caste) perform most of the other tasks such as feeding, foraging, nest building, defence, nursing, and the care of nestmates^14^. Thus, the reproductive success of a colony depends mainly on the workers' ability and flexibility to perform different tasks, as well as their proficiency to respond to external environmental changes and internal perturbation^17^.

*Vespula germanica* (Fabricius) (Hymenoptera: Vespidae), commonly known as “German wasp” or “yellowjacket”, is an eusocial wasp native to Europe and Northern Africa that in the last century has spread worldwide through a remarkable invasion process^18^. In invaded areas, *V. germanica* is considered a pest due to its negative impact on numerous economic activities, such as beekeeping and agriculture, as well as its powerful sting and high spread rates^19,20^. In Argentina, *V. germanica* has successfully established in forested and urban areas of the colder regions of the country such as Patagonia. In temperate regions, *V. germanica* has an annual colony cycle which begins in spring when a single mated queen establishes a nest after hibernation. Following emergence of first workers, the queen dedicates herself exclusively to oviposition and regulates activities within the colony, while the workers take care of the expansion, and maintenance of the nest. Colonies continue to grow until the summer when population peaks. Towards the end of summer, the colony begins to produce reproductive individuals (i.e., drones and gynes), which leave the nest to mate. During early fall, colonies and drones die and the fertilized gynes seek dry, sheltered places to hibernate until the following spring^21–23^.

*Vespula germanica* presents a marked task division with a few distinctive individuals conforming the reproductive caste (i.e., queens and drones) and a numerous worker caste of sterile monomorphic females. Workers may specialize in a subset of tasks according to their age (i.e., temporal polyethism). Generally, younger workers perform various tasks within the nest (maintenance, larvae feeding, and brood care) and older workers perform tasks outside the nest (foraging and nest defence)^23,24^. *Vespula germanica* is a scavenger and generalist wasp. Foragers consume carbohydrates to satisfy their own energy requirements and those of other nestmates, while protein foods are collected from dead mammals or insects for larval feeding^23,25^. Workers distribute food within the colony via trophallaxis. The larvae feed protein and regurgitate a saliva rich in carbohydrates and nutritive amino acids which are consumed by adult workers^23,26^. Foraging is essential for social insects because it allows colonies to grow (i.e., produce workers) and reproduce (i.e., produce reproductive individuals). However, one of the most important tasks workers need to perform to ensure colony success consists of the rearing and nursing of gynes^27^. In *Vespula* spp., gyne and drone production occur at the end of the summer season, as the colony as whole will die and only mated gynes will start a new colony after overwintering effectively^18,20^.

Our aim was to identify Allee effects and explore underlying mechanisms in *V. germanica* colonies through the experimental removal of workers. Our hypothesis is that there are component Allee effects in *V. germanica* colonies given that this wasp is a truly social species, with caste differentiation and cooperative tasks. The removal of workers affects the normal deployment of duties by colony members, ultimately affecting investment in reproduction. Specifically, we expected that the decrease in the number of workers in the colony leads to a decrease (a) in the number of gynes produced, (b) in the body mass index of gynes and (c) in the amount of protein-foods collected by foragers. Our research aims to enhance our understanding of the significance of Allee effects in social insects by examining their well-developed organization and cooperative behaviours, which effectively maximize their overall fitness.

## Materials and methods

**Figure 1:**
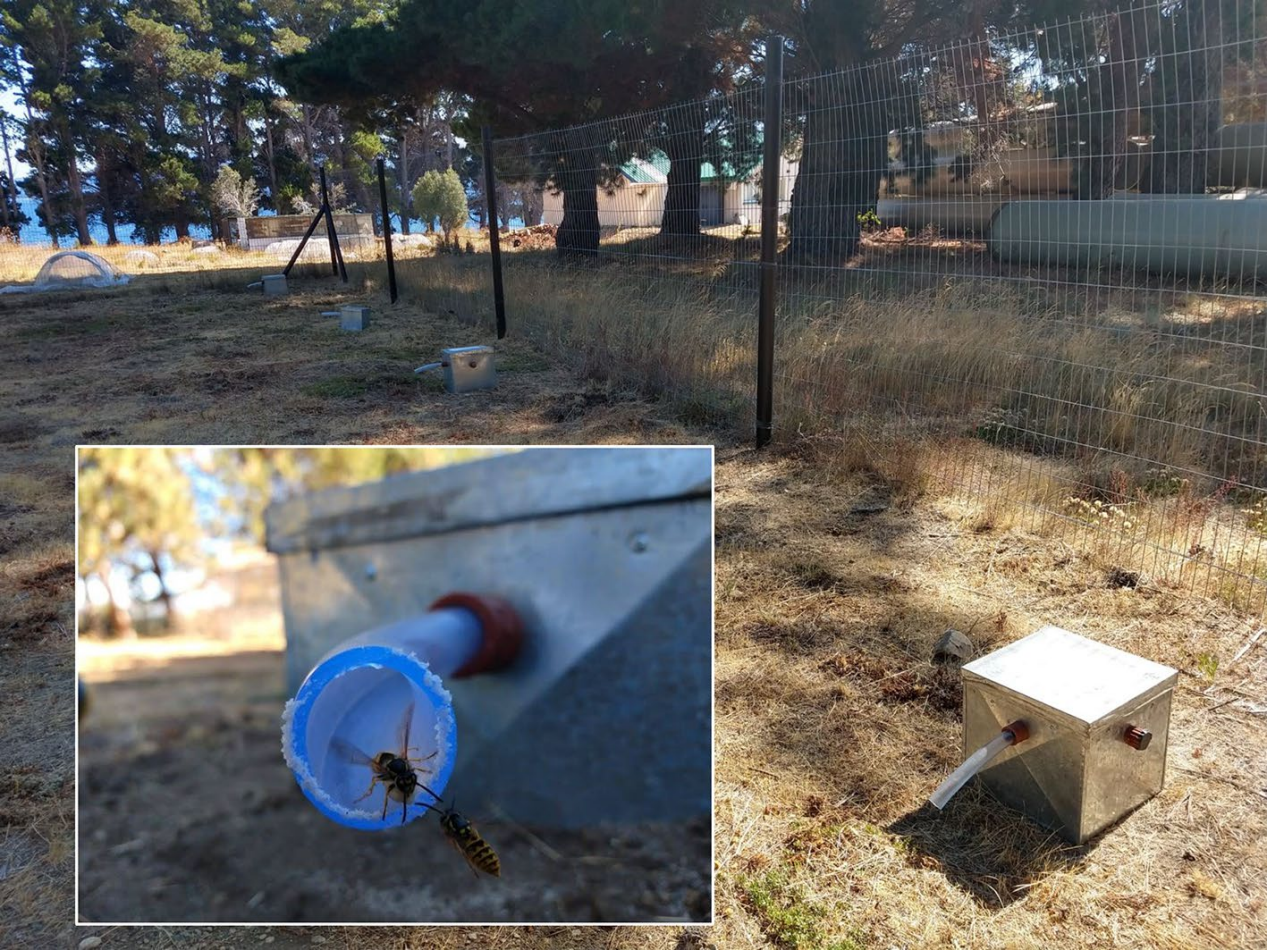
Nesting boxes within a fenced field site under natural ambient conditions. The nests are connected to the outside through a transparent plastic hose through which the workers enter and leave the nest. The workers quickly learn to use this new entrance, which allows the colony to function normally in the new location.

Twenty-two subterranean nests of *V. germanica* were collected in the vicinity of San Carlos de Bariloche, Argentina (41°09′S, 71°18′W) during February 2019 and March 2021 (11 nests each year). Once identified in the field, at a stage in which no reproductive individuals were present, nests were anesthetized with ethyl ether (98% purity; Sigma Aldrich, St. Louis, MO, USA) and carefully extracted from the soil in which they were buried. Nests were placed in individual nesting boxes (see below) and immediately transported to their final location, the grounds of IFAB (*Instituto de Investigaciones Forestales y Agropecuarias Bariloche*, Argentina). All nesting boxes were placed on the ground within a fenced field site under natural ambient conditions, 3 m apart from each other until the end of the experiments. Each box was constructed using zinc material with dimensions of 30 × 30 × 30 cm. The box was thermally insulated by applying high-density polyethylene foam, 2.5 cm thick, on its sides and base. The upper side of the box featured a double roof, an insulated layer, and a glass cover, providing the means to observe the development of the colony. On one side of the box, a tightly connected hole was present, which served as an entrance for wasps via a transparent plastic hose measuring 20 cm in length and 2.5 cm in diameter. This hose allows the workers to freely enter and exit the colony to forage and perform other tasks (Fig. 1). Previous studies showed that wasps rapidly adopted the nesting boxes and continue with their normal activities soon after removal from the field^28^.

After 5 days of acclimatization in their new location, nest traffic rate was measured (5 times a day for each nest) during five consecutive days during wasp peak activity hours (12 pm to 2 pm)^23^. Traffic rate was defined as the number of wasps leaving or entering the nest in one minute, which allowed us to estimate colony size using Malham’s formula (32.243 × traffic rate)^29^. The average of the maximum traffic of each day was used to determine the pre-treatment traffic rate (prior to worker removal). Nests were randomly assigned to one of two groups in both years: control (n = 3) and treatment (n = 8). Our treatments consisted in removing -once- different percentages of workers from the nests (from 0 to 80% approximately, as estimated from pre-treatment traffic rates) using a cord-free vacuum cleaner adapted for the task. The vacuum cleaner hose was introduced into the nest’s entrance to extract workers while ensuring no damage was made to nest structure. Only workers were removed thus maintaining the population of larvae, pupae, and eggs within each colony. The removal of workers was carried out at 6 am to ensure that most workers were inside the nest. Control nests were subjected to the same procedure but without removing any individuals, to standardize disturbance conditions. Following workers removal, traffic rate of each nest was measured again using the same methodology as that for pre-treatment traffic: five measurements per day for five consecutive days during wasp peak activity hours (12 pm to 2 pm). Post-treatment traffic rate was then calculated by averaging the maximum traffic recorded each day after worker removal. Subsequently, proportional change of workers (a shift in colony size), was calculated as (post-treatment traffic rate/pre-treatment traffic rate) × 100. Proportional change ranged from 24 to 146%, understanding that for proportional change values below 100%, the nest experienced a decrease in traffic rate and for values above, the opposite occurred (Table 1).

**Table 1:** *Vespula germanica* nests observed during 2019 and 2021 with pre-treatment traffic, post-treatment traffic, colony size^29^, the percentage of wasps removed, and the proportional change of workers for control (in bold) and treatment nests.

Year	Nest	Pre-treatment traffic (# workers/min)	Post-treatment traffic (# workers/min)	Colony size (# workers)	Worker’s removal (%)	Proportional change^a^ (%)
**2019**	**1**	**11.0**	**13.2**	**354.7**	**0**	**120**
	**2**	**16.8**	**19.4**	**541.7**	**0**	**115**
	**3**	**17.6**	**13.5**	**567.5**	**0**	**77**
	4	4.2	4.0	135.4	5	95
	5	10.8	8.6	348.2	20	80
	6	9.4	7.0	303.1	26	74
	7	16.6	9.4	535.2	43	57
	8	12.6	7.0	406.3	44	56
	9	22.6	11.0	728.7	51	49
	10	12.0	4.6	386.9	62	38
	11	9.0	2.2	290.2	76	24
**2021**	**12**	**7.0**	**10.2**	**225.7**	**0**	**146**
	**13**	**20.4**	**12.4**	**657.8**	**0**	**61**
	**14**	**14.6**	**11.6**	**470.7**	**0**	**79**
	15	21.6	20.6	696.4	5	95
	16	16.0	13.8	515.9	14	86
	17	20.4	15.6	657.8	24	76
	18	15.8	11.8	509.4	25	75
	19	13.4	8.6	432.1	36	64
	20	19.8	12.5	638.4	37	63
	21	11.0	6.8	354.7	38	62
	22	16.6	4.6	535.2	72	28

^a^Proportional change expressed as a percentage of pre-treatment traffic.

Protein foods gathered by workers were collected from foragers entering a nest during March to April of 2019 and 2021. One sample per day per nest was taken during 15 days in 2019, and 23 days in 2021 (i.e., n per nest 2019 = 15; n per nest 2021 = 23). To accomplish this, a cotton ball was inserted into the opening of the hose closest to the nest, and all workers attempting to enter were captured. After one minute, another cotton ball was used to seal the outer end of the tube containing the wasps. The hose was then removed and replaced with a new one to allow normal wasp activity. All wasps within the hose were anesthetized with carbon dioxide and food fragments were separated under a stereoscopic microscope (25×; Stemi 305, Carl Zeiss Microscopy GmbH, Germany) and weighed. Total food input per nest was calculated as the sum of the grams of food from all days sampled.

In order to assess whether worker removal affected colony fitness, all gynes produced by the nests were collected. For this purpose, daily during April and May (Austral fall) of 2019 and 2021, between 11 am and 4 pm, five observations of 10 min per nest were made to capture gynes naturally emerging from the nests^28^. Additionally, upon concluding the experiment in late May, we opened the colonies to capture any queens that remained inside the nests. The number of produced gynes per nest was recorded and everyone was weighed, and tibia length measured using a digital caliper (VWR^®^, Stainless Steel 0–150 mm, 0.01 mm resolution). The body mass index was calculated by dividing weight (mg) by tibia length (mm) (BMI = weight/tibia length).

### Data analysis:

To evaluate the effect of the proportional change on the number of gynes produced, a Generalized Linear Mixed Model (GLMM) with a Poisson error distribution was used. A GLMM with a log normal distribution was used for body mass index and Linear Mixed Model (LMM) with a normal error distribution for protein food collected. All models included proportional change as a fixed effect and colony size nested within year as random effects. Generalized Linear Mixed Models were fitted using *glmmTMB* function of the glmmTMB R package^30^ and LMM using *lmer* function of the lme4 R package^31^. Model validity was evaluated using the function *simulateResiduals* of the R package DHARMa^32^. All statistical analysis were performed with R version 4.0.2^33^.

## Results

All nests used survived until the end of the trials (i.e., late autumn) in both years in which experiments were conducted. For all treatment nests (n = 16), post-treatment traffic decreased after the removal of workers. The range of proportional change of workers for treated nests was from 24 to 95% with a mean of 63.87% ± 5.38% (mean ± s.e.). Furthermore, in control nests, the post-treatment traffic rate decreased in half of them (n = 3) after removal of workers, while it increased in the other 3. The range of proportional change within control nests was from 61 to 146%, with a mean of 99.67% ± 13.21%.

**Figure 2:**
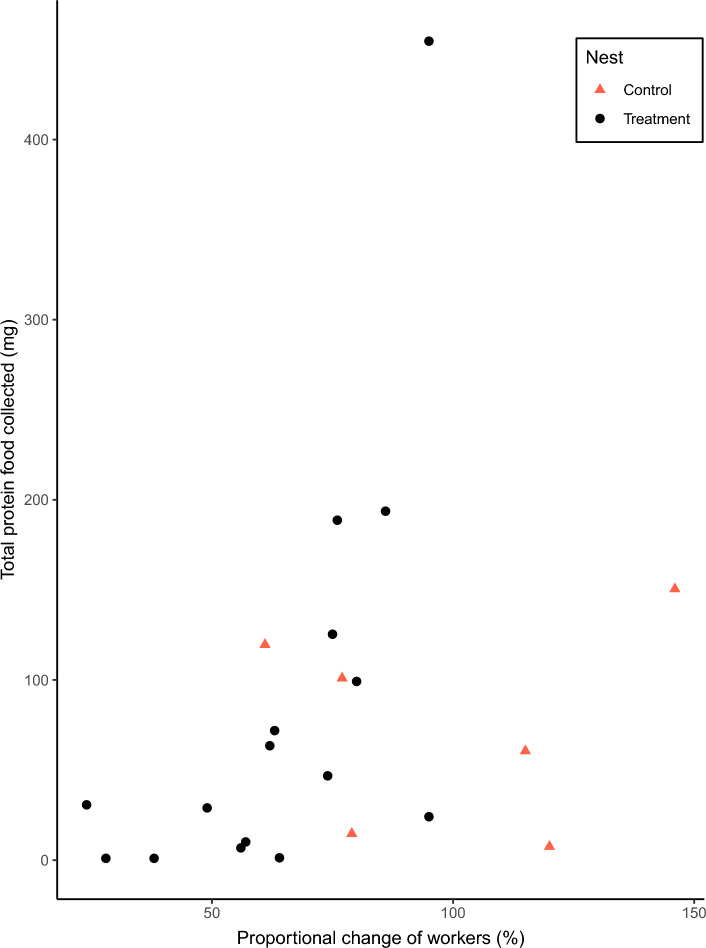
Total protein food collected per nest, for colonies with different proportional change of workers (post-treatment traffic expressed as a percentage of pre-treatment traffic). Red triangles indicate control nests and black dots treatment nests.

For protein food collected, no effect of the experimental reduction in the number of workers was observed (χ^2^_change_ = 3.64, d.f. = 1, P = 0.06, n = 22, Fig. 2). For controls nests, protein food collected varied from 7.5 to 150.5 mg (with a mean of 75.65 ± 23.64 mg, mean ± s.e.), while in treatment nests, varied from 1 to 454.6 mg (with a mean of 84.24 ± 29.28 mg, mean ± s.e.). Food samples taken from nests mainly consisted of whole arthropods or their parts, predominantly Arachnids, Coleoptera, Diptera, and other Hymenoptera, as well as fragments of animal protein. In addition to protein, wasps also consumed small amounts of plant material.

**Figure 3:**
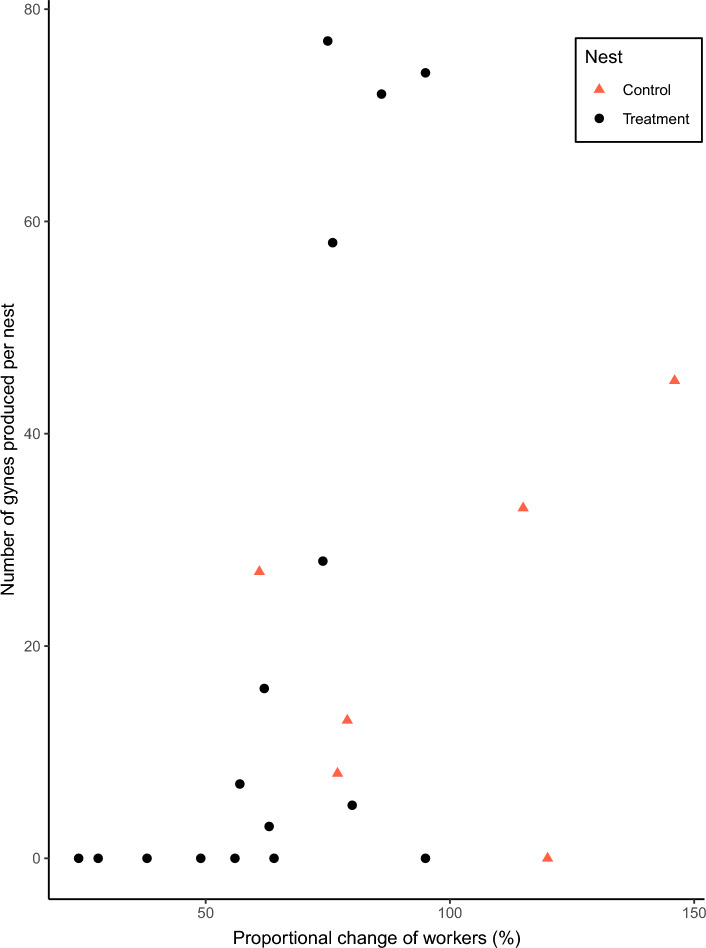
Number of *Vespula germanica* gynes produced per nest, for colonies with different proportional change of workers (post-treatment traffic expressed as a percentage of pre-treatment traffic). Red triangles indicate control nests and black dots treatment nests.

The number of gynes produced per colony significantly decreased as the proportional change of workers increased (χ^2^_change_ = 16.07, d.f. = 1, P = 6.09e^−05^, n = 22, Fig. 3). Control nests produced an average of 21.00 ± 6.90 gynes (mean ± s.e.), ranging from 0 to 45 gynes per nest. Only one nest did not produce any gynes during the study. Additionally, the number of gynes emerging from treatment nests ranged from 0 to 77 gynes, averaging 21.25 ± 7.59 gynes (mean ± s.e.). However, it is important to note that in nests where the number of workers had been reduced to less than 56% of the original number, no gynes were produced. (n = 7).

**Figure 4:**
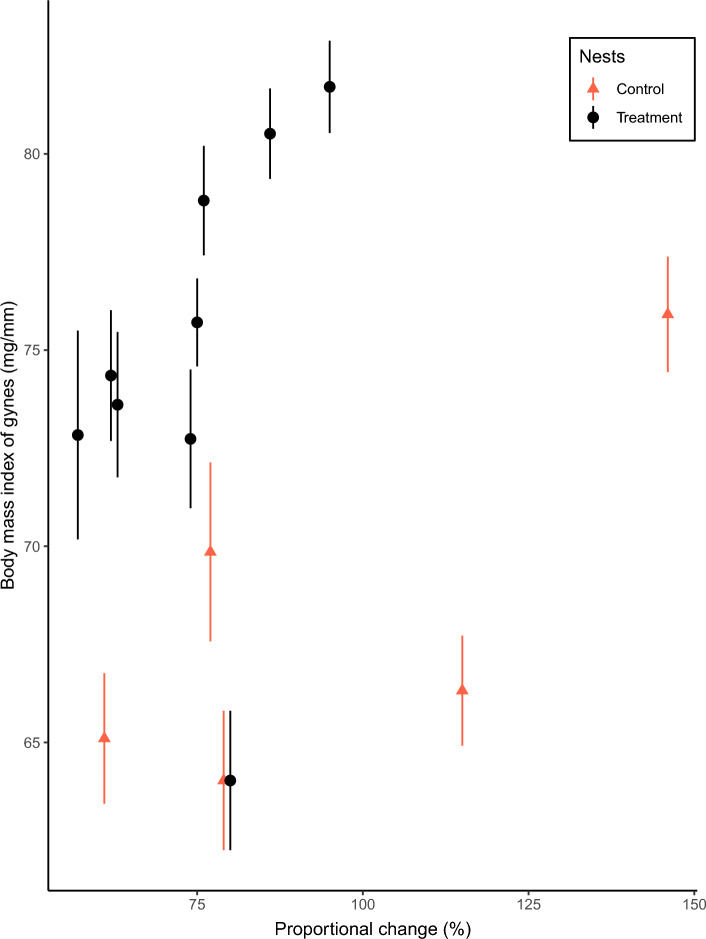
Body mass index of *Vespula germanica* gynes produced (mean ± s.e.) per nest, for colonies with different proportional change of workers (post-treatment traffic expressed as a percentage of pre-treatment traffic). Red triangles indicate control nests and black dots treatment nests. Triangles and dots denote median values, while whiskers standard errors.

Lastly, the body mass index of gynes was not affected by proportional change of workers (χ^2^_change_ = 2.22, d.f. = 1, P = 0.16, N = 22, Fig. 4). The body mass index of gynes from control nests ranged from 48.41 to 98.29 mg/mm, with a mean of 69.82 ± 0.91 mg/mm (mean ± s.e.). For treatment nests the body mass index ranged from 48.58 to 102.19 mg/mm, averaging 77.62 ± 0.54 mg/mm (mean ± s.e.).

## Discussion

This study assessed Allee effects in *V. germanica* wasps exploring possible components at the colony level. Our main finding was that with a decrease in the number of workers, colony reproduction was negatively affected. When workers reduction was important (reductions greater than 56% in worker traffic) no gynes were produced, suggesting that a minimum number of workers was needed to ensure colony reproduction. Such findings provide empirical evidence of Allee effects existence in this social wasp.

Success of truly social insects can be explained, in part, by the fact that related individuals actively cooperate in many tasks such as prey capture, feeding and brood care. The ability to execute these tasks is directly associated with increased colony productivity and survival^34^. In bees, the number of eggs successfully reared as larvae is dependent on both breeding and foraging individuals^35^. Also, it has been shown that in Argentine ants (*Linepithema humile*), queen productivity increases with worker abundance as workers are directly involved in rearing their brood^11,36^. *Vespula germanica* queens lay eggs while workers perform all other nest-related tasks, including brood care. To produce gynes, wasp colonies allocate more workers to tasks such as foraging and caring of the young^23^. Other factors such as nest expansion, queen cell construction, the maintenance of relatively high and constant nest temperatures and the availability of food, play an important role in colony survival and gyne production. Therefore, an abrupt decrease in the worker population within the colony is expected to negatively impact colony activities, which in turn might affect the investment in gyne production.

The fact that the removal of workers did not affect the amount of protein food taken into the nest may be explained by behavioural plasticity. This species uses protein food for feeding developing gyne larvae and cannot store it, thus workers need to make numerous and efficient foraging bouts to meet the demands of a growing colony, especially at the end of the summer season when investment in reproductives peaks^37,38^. Also, it is known that *V. germanica* workers could perform different tasks in a flexible manner^24,39^. Such a behaviour has been observed in other social insects, which adjust the number of workers performing specific tasks in response to environmental disturbances^40^. *Vespula germanica* displays a weak temporary polyethism due to the great variability in the sequence of task performance and in the variety of tasks accomplished by workers. Forty-three percent of the workers perform more than one task per day, which would indicate that colonies deploy a general strategy for the division of labour^24^. Particularly in temperate zones, where colonies have only one season to reproduce, *V. germanica* colonies use a general strategy for labour division that could enhance population growth and worker production^14,24^. This strategy could also be advantageous in mitigating the effects of a decrease in the number of workers by allowing colonies to rapidly compensate for the needed tasks. However, the removal of workers may affect other cooperative behaviours that contribute to colony fitness, such as nest hygiene and defence. Future research should investigate the impact of reducing the number of workers on the allocation of individuals to various tasks and how this, in turn, affects colony fitness.

Similarly, when a low proportion of workers were experimentally removed, there was no evidence of a decline in the quality of the produced gynes. In *V. germanica* as in other social insects, the differences between workers and gynes are mainly due to the quality and quantity of food they receive during their larval stage^23^. Gyne larvae are fed with food of a higher trophic level, or with a greater quantity of prey or excretions to meet their nutritional demands^41–43^. Protein intake was not affected by the decrease in the number of workers; this could potentially explain the similarity in body mass between queens from treated and control nests. It is possible that better gynes were produced at the cost of lower numbers, but this deserves further research.

Cooperative mechanisms that act at the colony level do not necessarily have to be the same as those affecting the population level^5,9^. The decrease in the number of gynes produced by colonies due to a decrease in the number of workers, may have implications for the establishment of new colonies in the following year. The success of a colony, including its growth in size and the number of workers, is closely related to the production of reproductive individuals. Given that queen mortality during winter is high (for example, for *V. vulgaris* it is known to be above 97.8%)^44^, chances that newly emerged queens can establish a colony successfully are limited. As colony foundation and initial survival are vital processes influencing the population level, it would be interesting to evaluate whether the decrease in the number of gynes ultimately leads to a reduction in the population growth rate of this invasive species over the long term.

To our knowledge, this study is the first research investigating the existence of Allee effects in colonies of an eusocial wasp. We found that a decrease in number of workers negatively affects gyne production while having no effect on gyne quality or protein intake. These results highlight the importance of cooperation between individuals of the same colony and contribute to our understanding of task assignment and plastic behaviours in social wasps. Despite limitations within our work, this research provides novel information to enhance our understanding of the social structure of social insects and support the theory of Allee effects in social insects.

## Data Availability

https://drive.google.com/drive/folders/1FEBovxxNtmp085RAf1UJaTwLojvMJm_A?usp=sharing.
